# The effects of Stemregen^®^ host modulation therapy on experimentally induced apical periodontitis in rats*

**DOI:** 10.1590/1678-7757-2024-0446

**Published:** 2025-02-03

**Authors:** Fatma GÖNÜLLÜ, Mevlüt Sinan OCAK, Serkan DUNDAR, İbrahim Hanifi ÖZERCAN

**Affiliations:** 1 Isparta Oral and Dental Health Center Isparta Turkey Isparta Oral and Dental Health Center, Isparta, Turkey.; 2 Firat University Faculty of Dentistry Department of Endodontics Elazıg Turkey Firat University, Faculty of Dentistry, Department of Endodontics, Elazıg, Turkey.; 3 Firat University Faculty of Dentistry Department of Periodontology Elazıg Turkey Firat University, Faculty of Dentistry, Department of Periodontology, Elazıg, Turkey.; 4 Firat University Faculty of Medicine Department of Pathology Elazıg Turkey Firat University, Faculty of Medicine, Department of Pathology, Elazıg, Turkey.

**Keywords:** Apical periodontitis, Stemregen^®^, Host modulation, Rat

## Abstract

This study evaluated the effect of Stemregen^®^ nutritional supplement on inflammation and resorption in apical periodontitis using a rat model.

**Methodology:**

Rats were divided in three groups: negative control (n=7), positive control (n=10), and Stemregen^®^ (Stem) (n=10). Apical periodontitis was induced in the positive control and Stem groups, and all rats were sacrificed on the 30^th^ day. Serum phosphorus (P), calcium (Ca), and alkaline phosphatase (ALP) were analyzed. Histopathological assessments measured osteoblastic and osteoclastic activity, inflammation, fibrosis, and abscess density. Immunohistochemical analyses evaluated RANKL, TRAP, and OPG levels.

**Results:**

Results showed significantly lower osteoblastic activity in the negative control compared to Stem and positive control groups (p=0.005). Osteoclastic activity was higher in the positive control (p=0.032). Inflammation and abscess formation were reduced in the Stem group compared to the positive control (p<0.001). OPG levels were lower in the negative control compared to the other groups (p=0.005).

**Conclusion:**

Stemregen^®^ effectively reduced inflammation and bone destruction, suggesting potential benefits for apical periodontitis management, though further research is needed.

## Graphical abstract

**Figure:**
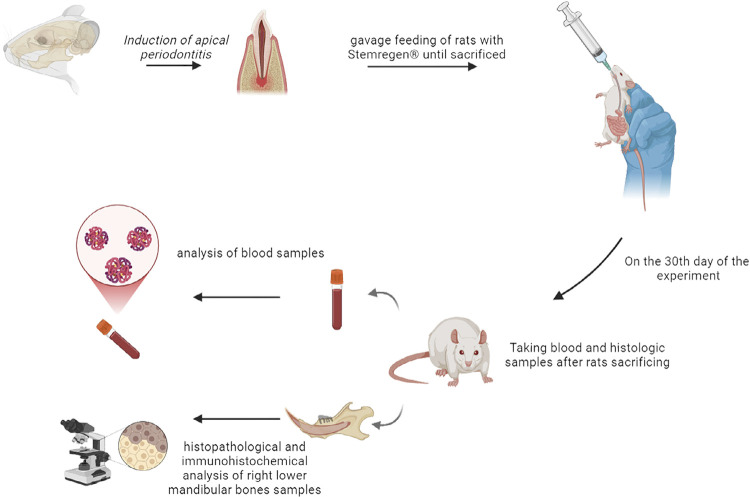

## Introduction

Apical periodontitis (AP) is an inflammatory disease that affects tissues surrounding the apical part of the root and is primarily caused by microorganisms infecting the root canal. In response to endodontic infection, host cells secrete inflammatory cytokines and activate cells associated with bone resorption, such as osteoclasts, leading to the demineralization of the surrounding bone tissue.^1^ Changes in periapical tissue are result of host defense reactions, including antigen-specific immunological responses following bacterial loading. Immunological responses during the formation of a periapical lesion can actively function to eliminate the invasion of exogenous antigenic substances.^2^

The purpose of host modulation therapy (host response modulation) is to achieve a balance between pro-inflammatory mediators and destructive enzymes on the one hand, and anti-inflammatory mediators and enzyme inhibitors on the other.^3^ Decontamination is the most common type of modulation. The logic behind this approach is to assist the host in its fight against infectious agents by supporting natural defense mechanisms or modifying the host’s response by altering the course of inflammatory systems. Compared to other approaches against infection, host response modulation has potentially fewer side effects, is non-invasive, and does not require complex methods of administration.^4^ Stem cell-based therapies, anti-inflammatory agents, growth/differentiation factors, and systemic or local applications of lasers can be used for host response modulation.^5^ Natural stem cell mobilizers can be effective tools for improving overall health and accelerating the healing process by supporting actual tissue repair and reducing inflammation.^6^

Stemregen^®^ is a food supplement that promotes stem cell migration and consists of blue-green algae (Aphanizomenonflos-aquae-AFA), prairie needle (Hippophaerhamnoides), bubbly algae (Fucus Vesiculous), Panax Notoginseng, Aloe Macroclada, transfer factor, and beta glucan. Studies have reported that the substances contained in Stemregen^®^ increase the number of circulating stem cells, reduce stem cell mobilization, and decrease pro-inflammatory cytokine levels.^6-13^ AFA is an excellent source of protein, polyunsaturated fatty acids, minerals, sterols, carotenoids and phycocyanins, all of which are well known for their antioxidant, anti-inflammatory and anticancer properties.^14^ In addition to exhibiting natural antioxidant properties, country spindle has been reported to prevent plasma lipid peroxidation, cytotoxicity and even DNA damage.^15^ The antioxidant effects of seaweed extracts have been confirmed both in vitro and in vivo.^16,17^Panax notoginseng has been shown to stimulate the production of stem cell factors from bone marrow and to support the mobilization of these cells to plasma by reducing the adhesion effect of adhesion molecules on bone marrow mesenchymal stem cells.^10^ It has been shown that the number of stem cells can increase up to 53% within two hours after the use of Aloe macroclada.^11^ It has been clearly demonstrated that bovine colostrum (transfer factor) can stimulate IL-6 and granulocyte colony-stimulating factor as well as growth factor production and induce stem cell activity *in vitro* and *in vivo* following oral supplementation.^18-20^ The ability of β-glucans to modulate the wound healing process and reduce scarring in mice has been reported.^21^

The aim of this study was to evaluate the effectiveness between host modulation therapy provided with Stemregen^®^ nutritional supplement, in addition to routine nutrition, and inflammation/resorption resulting from apical periodontitis.

## Methodology

This study was approved by the Local Ethics Committee of Fırat University Animal Experiments on March 22, 2021, with decision number 1452. It was carried out at the Experimental Research Center of Fırat University, and the Helsinki Declaration rules were strictly followed during the experiments. The rats used in the experiments were obtained from the Experimental Research Center of Fırat University. The study involved 27 male 300–350-gram Sprague Dawley rats aged between 0.5 and 1. The animals were cared for during the experiment under a 12-hour light and 12-hour dark cycle, at ambient conditions of 22–24°C, in rooms with free access to food and water, and a special ventilation system.

The number of samples in the study was calculated with the help of the G*Power package program. It was determined that a sample size of 10 in each group and 30 in total is required, assuming a 0.70 effect size for three groups with 90% power (β) and a 0.05 margin of error (α). It was referenced that 10 samples were present in each group.

The rats were randomly divided into three groups. The teeth of the subjects in the negative control (NC) group (n=7) were not subjected to any procedures. Anesthesia for all rats in the positive control (n=10) and Stemregen^®^ (Stem) (n=10) groups was provided with intramuscular injections of ketamine (5 mg/kg) and xylazine (45 mg/kg). Coronal pulp was exposed from the occlusal surface on the right mandibular first molars of the rats using a portable device with a spherical carbide milling cutter (Jet Carbide ¼; Kavo Kerr Group, Orange, CA, USA) with a diameter of 0.5 mm. After completing the cavity preparation, the patency of the coronal pulp was assessed using a #10 K-file (Dega-Videya, Guangdong, China), and the pulp was made irregular using the same file. The occlusal cavities were left open into the oral cavity until euthanasia. In the Stem group, a mixture of powdered Stemregen^®^ (Biomics™, USA) in capsules was administered orally via gavage at a rate of 300 mg/kg dissolved in saline solution every day for 30 days after apical periodontitis induction.^22^

On the 30th day of the experimental setup, anesthesia was provided to the subjects by intramuscular injection of 45 mg/kg ketamine hydrochloride and 5 mg/kg xylazine. Intracardiac blood was taken from the rats using 10 cc syringes and transferred to vacuum tubes containing a clot activator. The blood samples were then centrifuged at 3000 rpm for 10 minutes. The serum samples were analyzed for phosphorus (P), calcium (Ca), and alkaline phosphatase (ALP) values using an autoanalyzer device (Advia 2400 Siemens).

### Histopathological and immunohistochemical analyses

For histopathological and immunohistochemical analyses, the skin and subcutaneous dissection of the skulls of the sacrificed subjects were performed. The mandibular bones were removed and placed in 10% formaldehyde. After fixation in 10% formaldehyde, the tissues were treated with a decalcifying agent (OSTEOFAST 1, BIOGNOST^**®**^ Zagreb, Croatia) for 48 hours. The samples were then embedded in paraffin blocks, and sections with a thickness of four µm were taken. The sections were stained using the hematoxylin and eosin (H&E) staining technique and examined and photographed under a light microscope (Olympus, B53, Japan).

Inflammation, osteoclastic and osteoblastic activity, fibrosis, and abscess densities were semiquantitatively evaluated and scored between 0–3 in hematoxylin-eosin stained sections to analyze the inflammatory profile and assess the condition of the periapical region of the right first mandibular molar. The score values used in the evaluation are shown in Table 1.

**Table 1:** Histopathological andimmunohistochemical evaluation

	SCORE VALUE			
	None	Light Density	Medium Density	Heavy Density
H**istopathologicalanalysis**				
Osteoblast cells	0	1	2	3
Osteoclast cells	0	1	2	3
Inflamation	0	1	2	3
Fibrosis	0	1	2	3
Abscess	0	1	2	3
**Immunohistochemical analysis**				
OPG (+) cells	0	1	2	3
RANKL (+) cells	0	1	2	3
T**RAP (+) Cells**	**0**	**1**	**2**	**3**

For immunohistochemical analysis, paraffin-free tissue sections were stained with primary antibodies Osteoprotegerin(OPG) (Anti-OPG/Osteoprotegerin Antibody sc-390518, Santa Cruz Biotechnology, Oregon, USA), RANK Ligand(RANKL) (RANKL(G-1): sc-377079, Santa Cruz Biotechnology, Oregon, USA), and tartrate-resistant acid phosphatase (TRAP) (TRAcP (9C5) mouse monoclonal antibody, Cell Marque TM Tissue Diagnostics, USA) at a dilution of 1/200 using the Ventana XT automatic staining device. The stained sections were examined under a light microscope (Olympus, BX53-Japan). Scoring was performed based on the observed osteoblast and osteoclast densities, as indicated in Table 1.^23-26^

### Statistical analysis

The data was analyzed with the IBM SPSS v.22 package program. Descriptive data in the study are presented as n and % values for categorical data and as mean ± standard deviation values for continuous data. Chi-square analysis was used to compare the categorical variables. The normality of the data was assessed using the Shapiro-Wilk test. In the intergroup comparison of quantitative data, the One-Way analysis of variance(ANOVA) test was applied to parameters exhibiting a normal distribution. For post hoc analysis, the Tukey test was selected. The statistical significance level was set at p<0.05 for all analyses.

## Results

### Biochemical results

The results of the blood tests are presented in Table 2. There were no significant differences observed in serum calcium concentration and alkaline phosphatase activity among the groups (p>0.05). However, the plasma phosphorus concentration in the positive control group was significantly lower than that in the Stem and negative control groups (p<0.001).

**Table 2:** Group evaluation in terms of biochemical parameters

	Stem	Positive control	Negative control	p*
	Mean±SD	Mean±SD	Mean±SD	
ALP (U/L)	229.3±60,6	212.3±49.1	210.4±49.0	0.709
Ca (mg/dL)	10.5±0.3	10.4±0.4	10.7±0.2	0.147
P (mg/dL)	7.7±0.4^a^	6.9±0.3^b^	7.6±0.5^a^	**<0.001**

*The One Way ANOVA test was applied. ^a,b^: Group from which the difference originates

### Histopathological results

**Figure 1:**
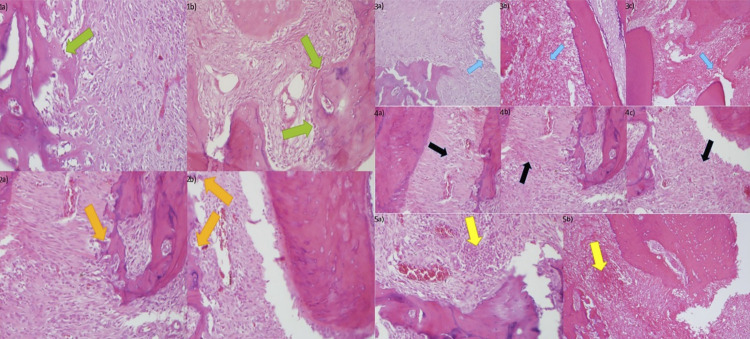
Sample images of the densities detected in the groups according to the scores in Table 1:1a) Score 1 osteoblastic activity, 1b) Score 2 osteoblastic activity, H&E (X400), 2a) Score 1 osteoclastic activity 2b) Score 2 osteoclastic activity, H&E (X400), 3a) Score 1 inflammation, 3b) Score 2 inflammation, 3c) Score 3 inflammation, H&E (x400), 4a) Score 1 fibrosis, 4b) Score 2 fibrosis, 4c) Score 3 fibrosis, H&E (x400) and 5a) Score 1 abscess, 5b) Score 2 abscess, H&E (x400).(Green arrow: osteoblastic activity, Orange arrow: osteoclastic activity, Blue arrow: inflammation, Black arrow: fibrosis and Yellow arrow: Abscess).

When the groups were analyzed according to osteoblastic activity, the negative control group had lower osteoblastic activity (p=0.008), and there was a significant difference between the groups in terms of mean osteoblastic activity (p=0.005) (Table 3). According to osteoclastic activity values, there was no significant difference between the groups in terms of osteoclastic activity (p=0.074), but there was a significant difference in terms of mean osteoclastic activity (p=0.032) (Table 4). There was a significant difference between the groups in terms of inflammation and mean inflammation (Table 5), fibrosis and mean fibrosis (Table 6), and abscess formation and mean abscess formation (Table 7) (p<0.001).The hematoxylin and eosin staining images of different scoring used in the histopathologic evaluation are shown in Figure 1.

**Table 3:** Group evaluation in terms of osteoblastic activity

		Stem		Positive control		Negative control		P
	Score	Number	%	Number	%	Number	%	
**Osteoblastic**	0	0	0	0	0	1	14.3	0.008*
**activity**	1	3	30.0	3	30.0	6	85.7	
	2	7	70.0	7	70.0	0	0	
**Median**		2^a^		2^a^		1^b^		0.005**
**Mean**		1.7^a^		1.7^a^		0.9^b^		

*Chi-square analysis, **The Kruskal Wallis analysis was applied. ^a,b^: Group from which the difference originates

**Table 4:** Group comparison according to osteoclastic activity

	Score	Stem		Positive control		Negative control		P
		Number	%	Number	%	Number	%	
**Ostaoclastic**	0	2	20.0	0	0	4	57.1	0.074*
**activity**	1	6	60.0	8	80.0	3	42.9	
	2	2	20.0	2	20.0	0	0	
**Median**		1^a,b^		1^a^		0^b^		0.032**
**Mean**		1.0^a,b^		1.2^a^		0.4^b^		

*Chi-square analysis, ** The Kruskal Wallis analysis was applied. ^a,b^: Group from which the difference originates

**Table 5:** Group comparison according to inflamation

	Score	Stem		Positive control		Negative control		P
		Number	%	Number	%	Number	%	
**Inflamation**	0	4	40.0	0	0	7	100.0	<0.001*
	1	5	50.0	4	40.0	0	0	
	2	1	10.0	5	50.0	0	0	
	3	0	0	1	10.0	0	0	
**Median**		1^a^		2^b^		0^a^		<0.001**
**Mean**		0.7^a^		1.7^a^		0^a^		

*Chi-square analysis, ** The Kruskal Wallis analysis was applied. ^a,b^: Group from which the difference originates

**Table 6:** Group comparison according to fibrosis formation

	Score	Stem		Positive control		Negative control		p*
		Number	%	Number	%	Number	%	
**Fibrosis**	0	0	0	0	0	6	85.7	**<0.001**
	1	7	70.0	2	20.0	1	14.3	
	2	3	30.0	7	70.0	0	0	
	3	0	0	1	10.0	0	0	
**Median**		1^a^		2^a^		0^b^		<0.001**
**Mean**		1.3^a^		1.9^a^		0.1^b^		

*Chi-square analysis, ** The Kruskal Wallis analysis was applied. ^a,b^: Group from which the difference originates

**Table 7:** Group comparison according to abcess formation

	Score	Stem		Positive control		Negative control		P*
		Number	%	Number	%	Number	%	
**Abscess**	0	8	80.0	1	10.0	7	100.0	**<0.001**
	1	2	20.0	6	60.0	0	0	
	2	0	0	3	30.0	0	0	
**Median**		0^a^		1^b^		0^a^		<0.001**
**Mean**		2^a^		1.2^a^		0^a^		

*Chi-square analysis, ** The Kruskal Wallis analysis was applied. ^a,b^: Group from which the difference originates

### Immunohistochemical results

**Figure 2:**
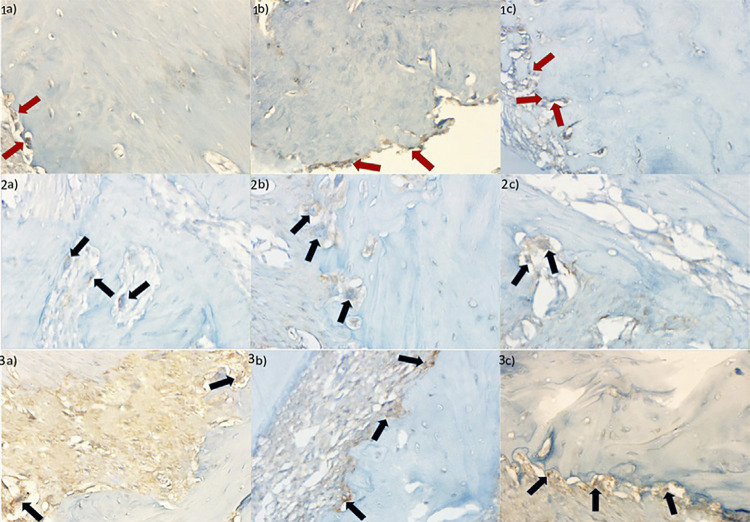
Sample images of the OPG, RANKL and TRAP: OPG (+) cells 1a) negative control group, 1b) positive control group, 1c) Stem group (x400), RANKL (+) cells 2a) negative control group, 2b) positive control group, 2c) Stem group (x400), and TRAP (+) cells 3a) negative control group, 3b) positive control group, 3c) Stem Group (x400).(Red arrow: OPG(+) cell, Black arrow: RANKL(+) cell in 2a, 2b, 2c, Black arrow: TRAP(+) cell in 3a, 3b, 3c).

There was a significant difference between the groups regarding OPG staining and mean OPG staining (p=0.008 and p=0.005, respectively) (Table 8). While no significant difference was observed between the groups for RANKL staining values (p=0.074), a significant difference was detected for the mean RANKL staining (p=0.032) (Table 9). There was no significant difference between the groups inTRAP staining (p=0.173) and mean TRAP staining (p=0.071) (Table 10). Images of the different scoring used in the evaluation of OPG, RANKL and TRAP are shown in Figure 2.

**Table 8:** Group comparison according to OPG

	Score	Stem		Positive control		Negative control		p
		Number	%	Number	%	Number	%	
**OPG**	0	0	0	0	0	1	14.3	0.008*
	1	3	30.0	3	30.0	6	85.7	
	2	7	70.0	7	70.0	0	0	
**Median**		2.0^a^		2.0^a^		1.0^b^		0.005**
**Mean**		1.7^a^		1.7^a^		0.9^b^		

*Chi-square analysis, ** The Kruskal Wallis analysis was applied. ^a,b^: Group from which the difference originates

**Table 9:** The comparison of groups according to RANKL

	Score	Stem		Positive control		Negative control		p
		Number	%	Number	%	Number	%	
**RANKL**	0	2	20.0	0	0	4	57.1	0.074*
	1	6	60.0	8	80.0	3	42.9	
	2	2	20.0	2	20.0	0	0	
**Median**		1.0^a,b^		1.0^a^		0^b^		0.032**
**Mean**		1.0^a,b^		1.2^a^		0.4^b^		

*Chi-square analysis, ** The Kruskal Wallis analysis was applied. ^a,b^: Group from which the difference originates

**Table 10:** Group comparison according to TRAP

	Score	Stem		Positive control		Negative control		P
		Number	%	Number	%	Number	%	
**TRAP**	0	2	20.0	0	0	3	42.9	0.173*
	1	5	50.0	7	70.0	4	57.1	
	2	3	30.0	3	30.0	0	0	
**Median**		1.0		1.0		1.0		0.071**
**Mean**		1.1		1.3		0.6		

*Chi-square analysis, ** The Kruskal Wallis analysis was applied

## Discussion

Host modulation therapy aims to reduce tissue destruction and promote protective responses by modifying the host’s immune response.^27^Certain types of functional foods have been shown to enhance the regenerative capacity of the body by stimulating immune cell pathways that activate stem cell niches and counteract oxidative stress and inflammation.^28-32^Stemregen^®^, a mixture of various extracts, has potential anti-inflammatory effects. Our study explores its impact on apical periodontitis. Despite the lack of previous rat studies on Stemregen^®^, our research contributes valuable insights. Comparison with probiotic studies revealed similar trends in ALP, calcium, and phosphorus levels. Stemregen^®^ showed potential in modulating inflammatory responses and bone resorption.

Various inflammatory mediators and cellular mechanisms play roles in bone destruction in periapical diseases. Bone formation and osteoblast activity can be examined using bone alkaline phosphatase, and bone formation and destruction can be demonstrated.^33^ Alkaline phosphatase is the most widely used marker for bone metabolism and it hydrolyzes pyrophosphate, allowing the deposition of hydroxyapatite crystals in newly synthesized osteoid tissues.^34-36^ Ca and P were evaluated biochemically in our study because they are regulators of bone growth and mineralization.^37^

No prior rat studies on Stemregen^®^ exist, making our research a significant addition to the literature. In comparison to a probiotic study by Cosme-Silva, et al.^38^ (2021), our study observed a higher mean ALP value in the Stem group, although statistically insignificant (p=0.709). Similarly, plasma calcium concentrations aligned with the findings of Cosme-Silva, et al.^38^ (2021), with no significant differences between groups (p=0.147). Notably, the positive control group had a significantly lower P value than the Stem and negative control groups (p<0.001).

Histopathological and immunohistochemical examinations allowed semiquantitative interpretation, revealing lower inflammation and abscess formation in the Stem group. Osteoclast scoring in the positive control group was significantly higher than in the negative control group, while the stem group showed a numerical decrease, though not statistically significant.

Dal-Fabbro, et al.^39^ (2021) examined the impact of excessive caffeine on rat apical periodontitis, noting heightened bone resorption and altered inflammatory patterns.Similarly, Cosme-Silva et al.^38^(2021)explored the effects of probiotic formula, observing modulation in inflammation and bone resorption. In our study, histopathological comparison revealed significantly higher inflammation in the positive control group than the Stem and negative control groups. The Stem group exhibited lower inflammation, attributed to the antioxidant and anti-inflammatory properties of its plant extracts.^6-13^The favorable effects of plant extracts in Stemregen^®^ on inflammation are attributed to the mobilization of stem cells leading to a reduction in inflammatory cell infiltration and a reduction in various pro-inflammatory cytokines.^8,37^

Gatej, et al.^40^ (2018) studied probiotics impact on alveolar bone loss, noting increased inflammation and osteoclastic scores in the periodontitis control group compared to healthy controls. However, animals treated with probiotics showed scores similar to healthy controls. In our study, histopathological examination revealed significantly higher osteoclast scoring in the positive control group than the negative control group. Although the stem group exhibited a numerical decrease, there was no statistically significant difference from the positive control group.

In terms of fibrosis, the negative control group exhibited a significantly lower level than the positive control and Stem groups. The stem group numerically lower fibrosis may be linked to Stemregen^®^ increasing circulating stem cells, potentially influencing the healing process.

Dal-Fabbro et al. found excessive caffeine increased RANKL expression in apical periodontitis, confirmed by TRAP (+) cells.^39^Liu, et al.^41^(2011) reported the role of caffeine in osteoclastogenesis and decreased bone mineral density. Metformin, suggested to reduce RANKL/OPG ratio, inhibits osteoclasts.^42^Cosme-Silva et al.^38^ (2021) found that probiotic supplementation lowered RANKL levels and TRAP (+) cell count.

In our study, RANKL(+) cell density was significantly lower in healthy controls than positive controls with apical periodontitis. Stem group TRAP(+) cells were numerically lower than positive controls, with a potential shift towards osteogenesis suggested by higher RANKL expression in positive controls compared to the Stem group, despite stable OPG levels.

Our study indicates promising therapeutic potential for Stemregen^®^ in apical periodontitis. This effect is attributed to Stemregen^®^’s plant extracts, showcasing radical scavenging, antioxidant, anti-inflammatory properties, and the ability to enhance stem cell mobilization. However, diverse extract composition, their individual and combined use, mixture ratios, dosage, frequency, experimental models, and immune response modulation in rats present limitations. Although the characteristics and development of periapical lesions have been investigated in various studies, the effects of host modulation on periapical lesions have been investigated in a rat model study using Stemregen^®^. The rat model was preferred for this study because the development of apical periodontitis in rats reproduces the human healing process, allowing the histological examination of periapical tissues and standardization of specimens.^43^ Further studies are essential to translate our findings to human clinical applications. We anticipate that the data obtained from our study will provide guidance for new topics that researchers can study by providing host modulation with nutritional supplementation in addition to routine root canal treatment in patients with periapical lesions.

## Conclusions

As the first study to examine the effects of Stemregen^®^ on experimental apical periodontitis in rats, our study provides a potential foundation for future host modulation procedures aimed at reducing the extent of periapical disease destruction and promoting post-treatment recovery. Based on our findings, it can be inferred that by reducing the number of osteoclasts and modifying the RANKL/OPG ratio in apical periodontitis, it may be possible to suppress bone resorption and slow the progression of periapical disease.
